# Novel laparoscopic surgery for the repair of cesarean scar defect without processing scar resection

**DOI:** 10.1186/s12884-021-04281-8

**Published:** 2021-12-08

**Authors:** Ning-Ning Zhang, Guang-Wei Wang, Na Zuo, Qing Yang

**Affiliations:** Department of Obstetrics and Gynecology, Shengjing Hospital of China Medical University, Shenyang, 110004 China

**Keywords:** Cesarean section, Laparoscopy, Cesarean scar defect, Minimally surgical treatment

## Abstract

**Background:**

Cesarean scar defect (CSD), especially CSD with residual myometrium less than 3 mm is reported to be the highest risk agent associated with uterine rupture for subsequent pregnancy. Currently, laparoscopic resection and suture was the mainstay therapy method for CSD with a residual myometrium less than 3 mm in women with a desire to conceive. Besides, the women have CSD related symptoms, especially postmenstrual bleeding, should be recommended for CSD treatment. This study is to investigate the efficiency of this novel laparoscopic surgery for the repair of cesarean scar defect (CSD) without scar resection for residual myometrium thickening.

**Method:**

This retrospective clinical study enrolled 76 women diagnosed with CSD who had a residual myometrium thickness less than 3 mm and also had a desire to conceive, had undergone laparoscopic surgery for the repair of CSD in the time period March 2016 to March 2018. Two study cohorts were created among the 76 patients: 40 patients had undergone the novel laparoscopic repair of CSD without processing scar resection (Group A), whereas 36 patients had undergone the traditional laparoscopic resection and suture of CSD (Group B).

**Results:**

Residual myometrium thickening occurred among all the 76 patients and the average residual myometrium thickness was increased to almost 6 mm, presenting no between-group difference. In Group A, all the CSD-related postmenstrual bleeding was resolved or improved, but one patient in Group B has no obvious change to postmenstrual bleeding. After CSD repair, 20 patients got pregnant naturally in Group A, and there was no cesarean scar pregnancy and uterine rupture. While, there were 9 cases of natural pregnancy in Group B. No uterine rupture occurred among these 9 pregnant women of Group B, but 1 case of pregnancy was terminated due to cesarean scar pregnancy.

**Conclusion:**

Laparoscopic repair without processing scar resection seems to be a feasible, safe and simple operative approach for CSD treatment, which can thicken residual myometrium and improve postmenstrual bleeding.

## Background

Cesarean scar defect (CSD) is a serious long-term complication of cesarean section [1, 2]. Once CSD develops, the defect will disrupt the integrity of the myometrium at the site of the cesarean scar, resulting in a series of clinical symptoms, such as prolonged menstruation, postmenstrual bleeding, dysmenorrhea and so on [3–6]. The women have CSD related symptoms, especially postmenstrual bleeding, should be recommended for CSD treatment [7]. Additionally, CSD can cause obstetric complications as well, such as cesarean scar pregnancies and/or uterine rupture [8–10]. CSD with residual myometrium less than 3 mm is reported to be the highest risk factor associated with uterine rupture for subsequent pregnancy [11].

Laparoscopic scar resection and scar reconstruction to thicken the residual myometrium can be considered to be the mainstay therapy method for CSD with a residual myometrium thickness less than 3 mm in women who has a desire to conceive [12–17]. During resection and scar reconstruction, it needs to suture the two edges of uterine wall with different thickness after scar resection [12, 13, 18], and another newly formed scar defect should be considered due to the uncertain scar healing process, although less likely, possibility that a reservoir-like defect again located at the site of the scar [19]. Through carefully investigating the operation process and perioperative outcomes of surgical resection and suture of CSD, we conducted a novel operation approach to tactically repair the scar defect: laparoscopic repair the CSD without processing scar resection. In this study, we sought to compare two homogeneous groups of patients undergoing minimally surgery for CSD via laparoscopic repair without and with processing scar resection. Our secondary endpoint was to investigate long-term effectiveness of this novel laparoscopic surgery for the repair of CSD with retention of the integrated cesarean uterine scar.

## Methods

### Study design

This retrospective study enrolled 76 women diagnosed with CSD who fulfilled the criteria of this study at Shengjing Hospital of China Medical University in the time period March 2016 to March 2018. This study was approved by the Institutional Ethics Committee of Shengjing Hospital of China Medical University, and a written informed consent was obtained from all participants.

Women who presented with postmenstrual bleeding (postmenstrual bleeding was defined according Vervoort et al. study [11], as either ≥2 days of intermenstrual bleeding, or ≥ 2 days of brownish discharge immediately at the end of the menstrual period when the total period of the menstrual bleeding exceeded 7 days), and in whom preoperative transvaginal ultrasonography and magnetic resonance imaging had shown a defect with a residual myometrium thickness less than 3 mm, were eligible. All the included patients in final analysis had a desire to conceive in future and had no contraindications to surgery.

Exclusion criteria included being under the age of 18 years, pregnancy and lactation, infertility (including infertility and/or infertility treatment in the prior child and the subsequent fertility), irregular uterine bleeding caused by some other diseases, an irregular cycle (> 35 days or with an intercycle variation of 2 weeks or more), severe heart and lung diseases, liver and kidney impairment, and other severe underlying diseases, systemic infections or severe local infections.

Data were retrieved from computerized patient records alongside health declarations from clinic databases. Every participating patient’s record was assessed individually. The baseline characteristics including age, number of caesarean sections, postmenstrual bleeding, and residual myometrium thickness of CSD were registered.

### Surgical method

#### CSD confirmation

**Fig. 1:**
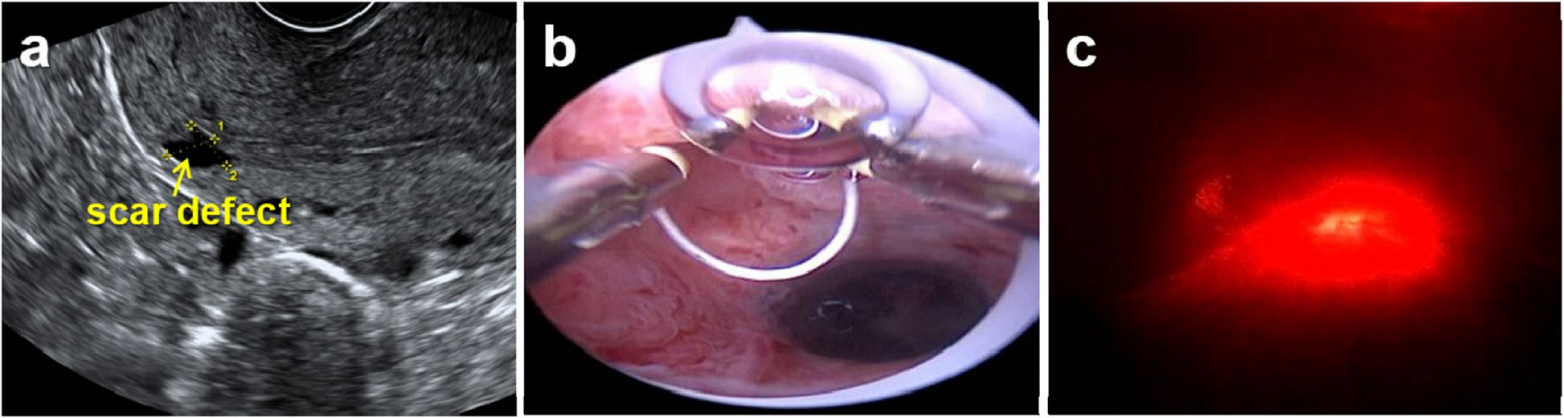
CSD examination and confirmation: **a** transvaginal ultrasound image; **b** hysteroscopic examination; **c** light test

Before laparoscopic surgery, all patients were examined using transvaginal ultrasound (Fig. 1a) and magnetic resonance imaging to identify the CSD. The CSD repair by laparoscopic surgery was guided by hysteroscopy (Fig. 1b) and light test (Fig. 1c) to confirm the upper and lower margins of the scar defect.

#### CSD repair

**Fig. 2:**
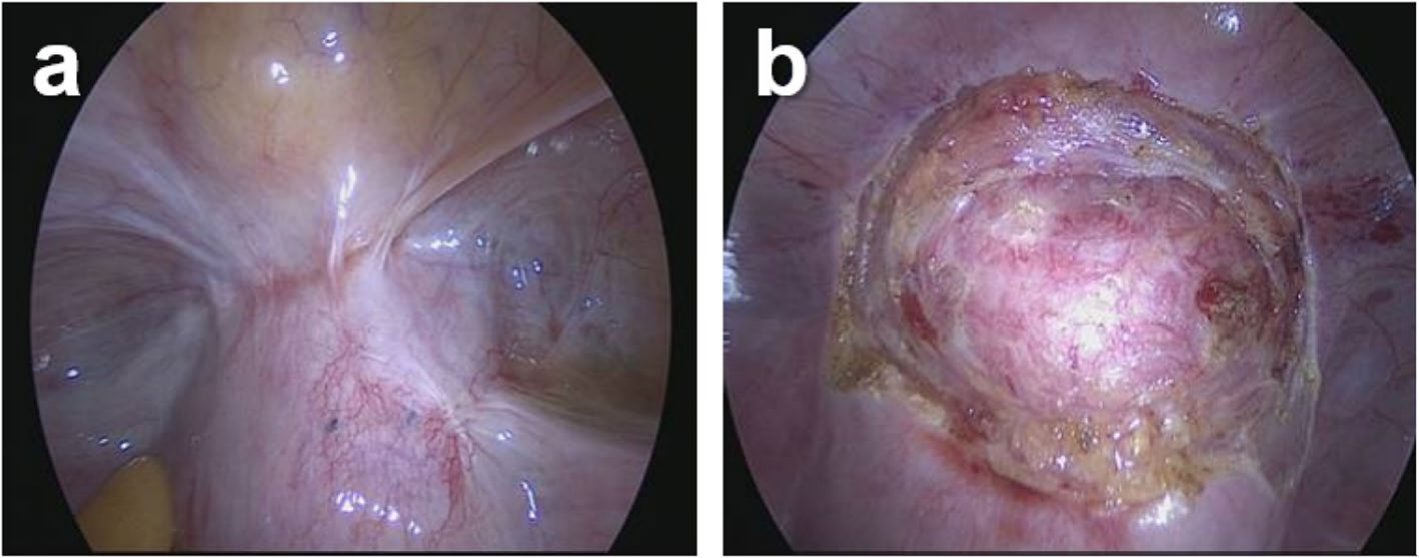
**a** Laparoscopic examination; **b** isolate adhesion between the bladder and anterior wall of uterine cesarean section scar

**Fig. 3:**
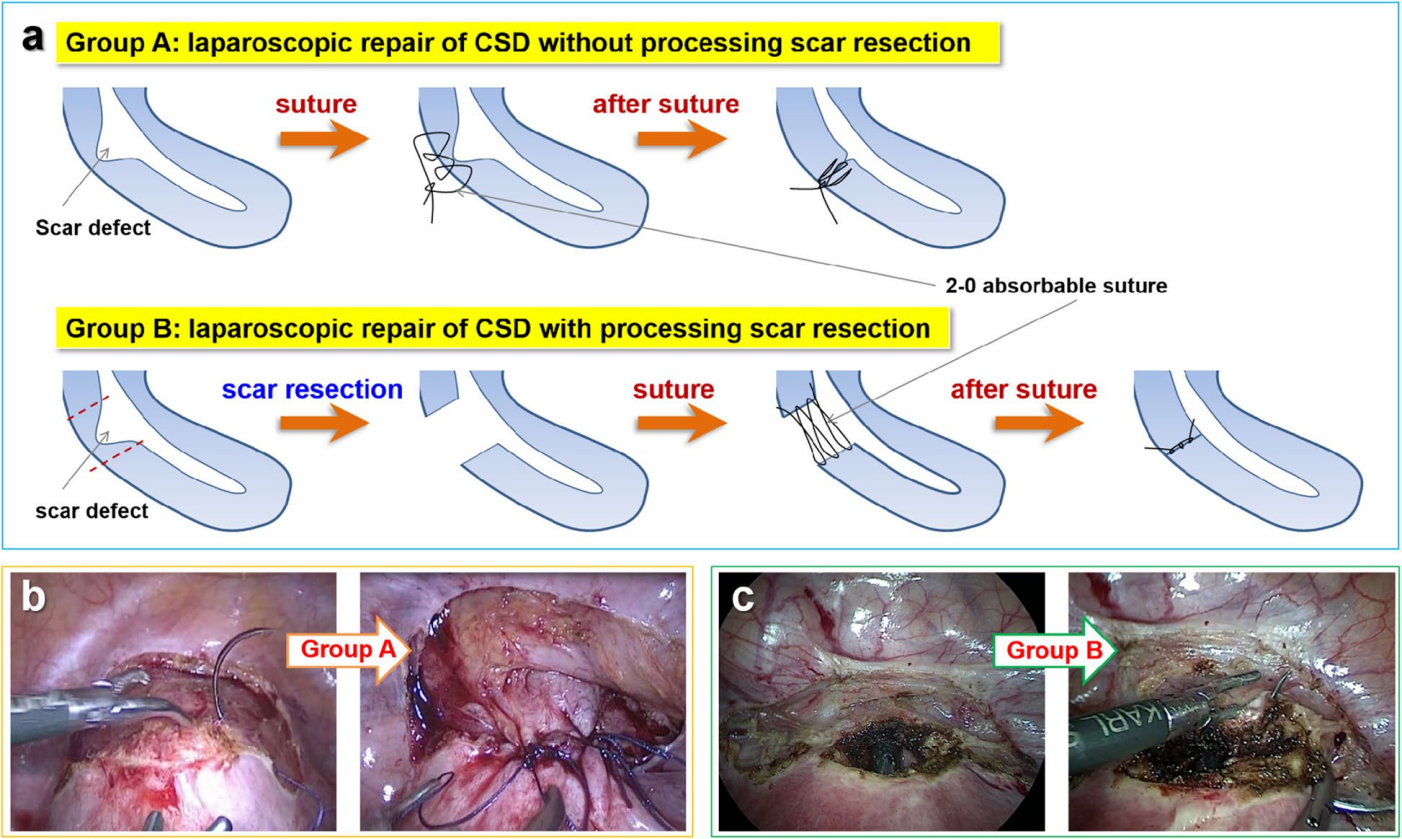
**a** A schematic overview of the method of laparoscopic repair CSD without (Group A) and with (Group B) processing scar resection; **b** laparoscopic suture upper and lower margin of the muscle layer in Group A; **c** laparoscopic excision of uterine scar defect and suture of the myometrium and serosa in Group B

Firstly, all patients were examined with laparoscopy to isolate adhesion between the bladder and anterior wall of uterine cesarean section scar (Fig. 2a and b). Then the patients underwent laparoscopic repair of CSD without (Group A) and with (Group B) processing scar excision. A schematic overview of the two methods was provided in Fig. 3a, which might be easier to grasp the surgery procedure with some visual aids.

In group A, in some cases, hysteroscopy was performed to coagulate the surface of CSD when the abnormal blood vessels were found. Otherwise, if there was no abnormal blood vessel found on the surface of CSD it did not need processing hysteroscopic coagulation. After that, a 2–0 absorbable suture was used to interruptedly appose and suture the upper and lower margins of the scar defect (Fig. 3a and b) to remove or reduce the size of the defect. Meanwhile, part of muscle tissue along the upper and lower margins of the scar was sutured into the scar defect to fill its weakness position, thus increasing the thickness of the residual myometrium. Then the serosa was followed by suture. As for Group B, patients were treated via uterine scar defect resection under laparoscopy, and then a 2–0 absorbable suture was used to sequentially suture the myometrium, and then serosa (Fig. 3a and c).

#### Checkup of CSD repair

**Fig. 4:**
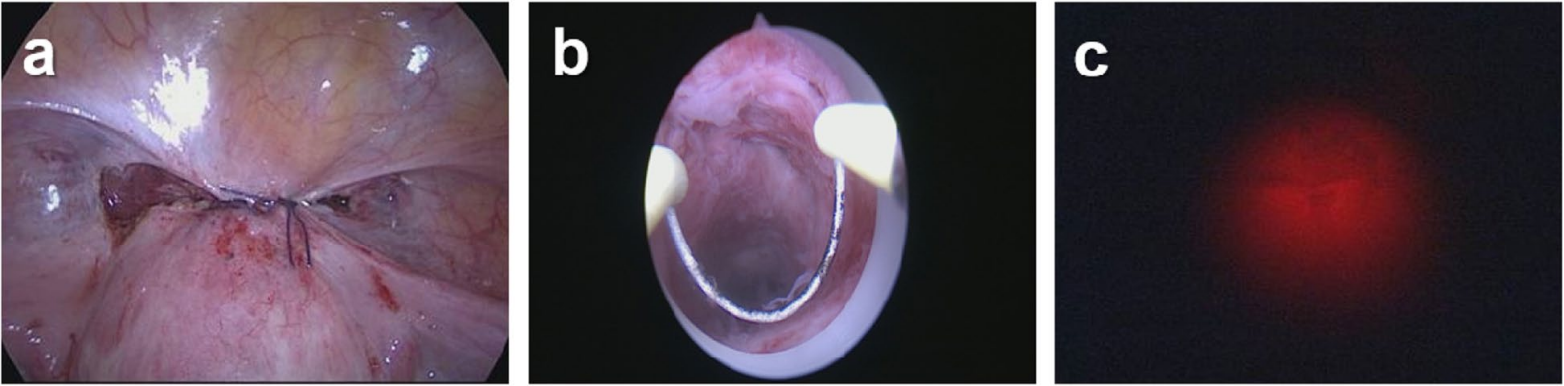
Checkup of CSD repair: **a** suture of the uterine serosa and bladder peritoneum; **b** hysteroscopy examination; **c** light test

As seen in Fig. 4, hysteroscopy and light test were used again to confirm the well repair of the defect. Meanwhile, intraoperative ultrasound was performed to measure the thickness of the residual myometrium. Finally, hemostasis, flushing, and abdominal drainage placement were carried out among all the patients.

### Core outcome sets

The main outcome measure was the efficacy of the treatment of CSD. At the 3-month and 12-month follow-up evaluation after surgery, enlargement of the residual myometrium thickness and reduction of postmenstrual bleeding were measured. The second was subsequent fertility outcome.

### Statistical analysis

SPSS 24.0 software was used for statistical analysis (t-test and χ^2^ test) with a *P* < 0.05 considered statistically significant. χ^2^ test was used for detailed residual myometrium and postmenstrual bleeding groups before and after CSD repair and surgical complications. t-test was used for the other data.

## Results

During the time period March 2016 to March 2018, a total of 76 women diagnosed with CSD who fulfilled the criteria of this study had undergone laparoscopic surgery for the repair of CSD. Among the 76 patients, 40 women were treated by the novel laparoscopic repair of CSD without processing scar resection (Group A), and 36 women underwent traditional laparoscopic repair of CSD with processing scar resection (Group B). The patient age was ranged from 26 to 40 years. Of the 76 patients, 71 underwent one cesarean section each, accounting for 93.4%, and other 5 patients underwent two cesarean sections. CSD-related characteristic of study patients was list in Table 1 and there were no differences between the two group. The 76 patients reported with postmenstrual bleeding for ca. 5 days (range: 3–10 days) and residual myometrium of ca. 2.0 mm (range: 1.2–2.8 mm). After CSD confirmation, the uterine scar defect was repaired under laparoscopy and the detailed surgery procedure of the two groups were shown in surgical method section and Fig. 1. The mean operation time of Group A was 55.85 ± 10.07 min (range, 46–85 min), which experienced shorter time than Group B (71.50 ± 5.94 min (range, 59–90 min); *p* < 0.001). There were no surgical complications in the two groups.

**Table 1:** Baseline and surgical outcome characteristics^&^

parameter	Group A (*n* = 40)	Group B (*n* = 36)	*P* value
**Baseline characteristics**			
Postmenstrual bleeding (days)	5.38 ± 1.67	5.25 ± 1.65	0.744
Residual myometrium (mm)	2.03 ± 0.43	2.00 ± 0.41	0.757
**Surgical outcomes**			
Operative time (min)	56.10 ± 10.90	71.08 ± 6.26	< 0.001
Surgical complications	0	0	1.0

^&^Values are means ± standard deviation

**Fig. 5:**
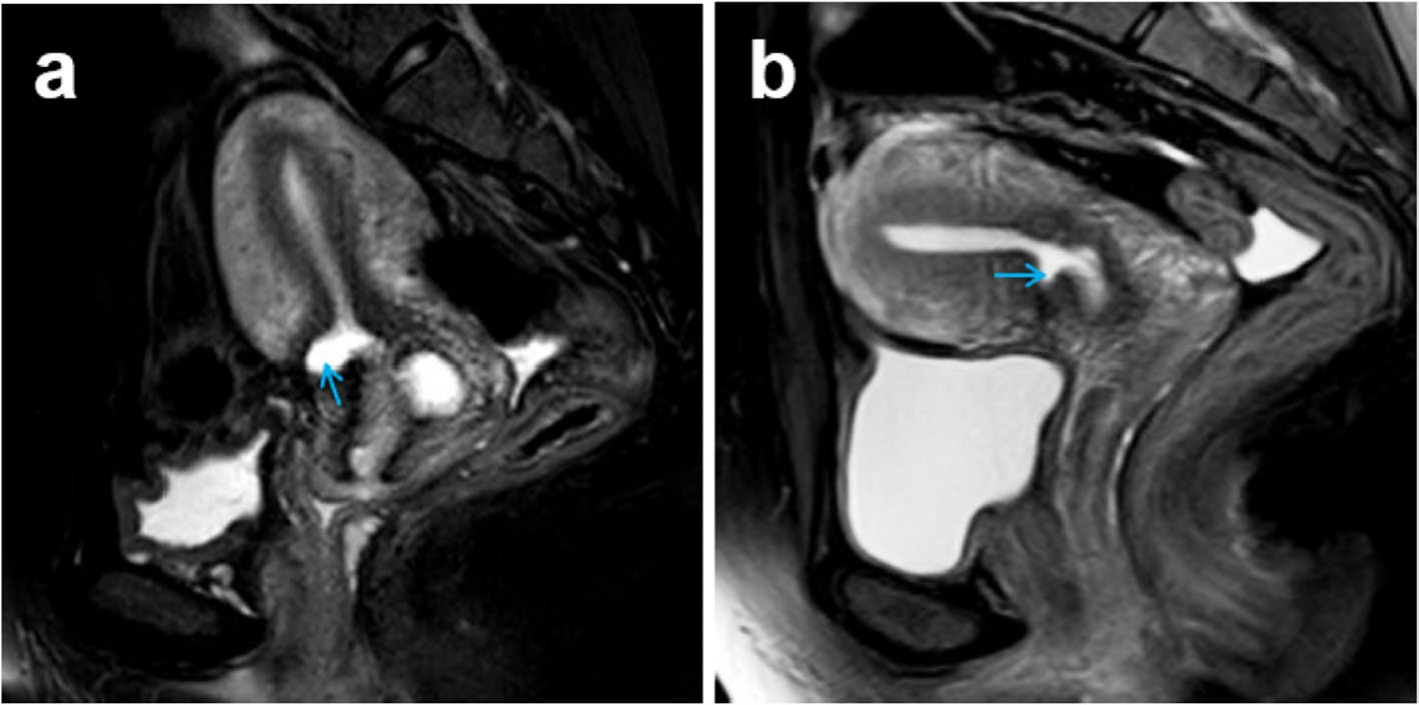
Magnetic resonance imaging result: **a** before CSD repair; **b** after CSD repair

Almost all the patients followed up for 3 to 12 months had satisfied outcome after CSD repair. The menstrual recovery and postmenstrual bleeding were reviewed, and the presence and any changes of defect, compared with the preoperative status, were confirmed (Table 2). At the 3rd month follow-up period, in Group A, all the 40 patients’ postmenstrual bleeding was reduced and 37 patients shortened postmenstrual bleeding to 0 day. However, at the 3rd and 12th month after CSD repair, one patient in Group B has no obvious change to postmenstrual bleeding and 31 patients have 0 day of postmenstrual bleeding. The average thickness of residual myometrium in all the patients was increased to almost 6 mm, presenting no between-group difference. As seen in Fig. 5, magnetic resonance imaging results show that the scar defect is reduced and the thickness of residual myometrium was substantially enlarged after laparoscopic suture CSD without processing scar resection (patient from Group A at the 3rd month). At the time of 12th month after CSD repair in Group A, excluding 4 pregnancies, postmenstrual bleeding of 35 patients was shortened to 0 day and the average thickness of residual myometrium in all the patients was increased to almost 6.2 mm (range: 4.3–7.8 mm). In group B, there is one patient still has with no obvious change to postmenstrual bleeding and no enlargement of residual myometrium, and the average thickness of residual myometrium of the 36 patients of Group B was increased to about 6.1 mm (range, 1.8–7.9 mm). After CSD repair, 20 patients got pregnant naturally in Group A, and there was no cesarean scar pregnancy and uterine rupture. Among the 20 pregnant women, 2 women had spontaneous miscarriage at early pregnancy, 2 women gave live premature at 32 ~ 34 weeks and the other 16 women gave live delivery at 37 ~ 40 weeks. While, there were 9 cases of natural pregnancy in Group B. No uterine rupture occurred among these 9 pregnant women, but 1 case of pregnancy was terminated due to cesarean scar pregnancy, and the other 8 16 women gave live delivery at full-term.

**Table 2:** 3-month/12-month follow up outcome characteristics^$^

parameter^#^	Group A (*n* = 40/*n* = 36)	Group B (*n* = 36/*n* = 36)	*P* value
**postmenstrual bleeding (days)**	**0.15 ± 0.58/0.06 ± 0.33**	**0.36 ± 1.05/0.17 ± 0.85**	**0.289/0.466**
0 day	37 (92.5%)/35 (97.2%)	31 (86.1%)/34 (94.4%)	0.365/0.555
0 day < after surgery < before surgery	3 (7.5%)/1 (2.8%)	4 (11.1%)/1 (2.8%)	0.587/1.000
after surgery ≥ before surgery	0 (0.0%)/0 (0.0%)	1 (2.8%)/1 (2.8%)	0.289/0.314
**residual myometrium (mm)**	**6.02 ± 0.80/6.18 ± 0.75**	**5.93 ± 1.01/6.09 ± 1.01**	**0.677/0.692**
after surgery > before surgery	40 (100%)/36 (100%)	35 (97.2%)/35 (97.2%)	0.289/0.060
after surgery ≤ before surgery	0 (0.0%)/0 (0.0%)	1 (2.8%)/1 (2.8%)	0.289/0.060

^$^ Values are means ± standard deviation

^#^ in Group A, 40 women were followed up at the 3rd month and 36 women were followed up at the 12th month, as 4 women got subsequent pregnancy at the time of the 12th month after CSD repair; in Group B, there was no pregnancy at the 12th month, hence, there were 36 patients for both at the 3rd and 12th month

## Discussion

CSD is a common complication of cesarean section delivery [1, 20]. Up to now, the specific cause of CSD is not completely clear; and it is believed that any factors interfering with uterine incision, such as the surgical opportunity, suture method, individual immune resistance and so on, can lead to different degrees of scar defect [2]. Most clinical studies suggest that CSD treatment should be performed on patient who has the clinical symptoms, especially postmenstrual bleeding [7, 11]. Florio et al [21] reported that the scar defect was an anatomic change; hence the symptoms could be relieved by surgical resection.

Currently, repair of CSD with residual myometrium thickness less than 3 mm usually involves surgery to remove the uterine scar at the site where defect forms, and then suture the normal uterine tissue together [22, 23]. Such scar reconstruction via scar resection followed by suture can increase the residual myometrium thickness which may reduce the risk of subsequent pregnancy. However, it’s still possible to form a new scar after the scar reconstruction.

The present study investigated the efficiency of the novel laparoscopic surgery without processing scar resection for the management of CSD. The difference between this study and traditional laparoscopic scar resection is that the cesarean uterine scar does not need to be resected in the former and it need to be resected in the later. From the anatomical basis view, the abnormal uterine bleeding happens for two reasons: muscular defect of the uterus scar and hyperplasia of abnormal blood vessels on the surface of some CSD, hence the novel laparoscopic surgery is mainly treating the above mentioned anatomical changes, namely remove the cesarean scar defect (increase local muscle layer thickness) and remove the hyperplasia of abnormal blood vessels (if the hyperplasia of abnormal blood vessels was found under hysteroscopy). Comparing the surgery procedure, laparoscopic repair of CSD without processing scar resection can retain the integrity of the cesarean uterine scar, which avoids newly formed scar. The scar was not reconstructed in this novel method, it was repaired. Patients could try to conceive when the symptoms are improved. Furthermore, laparoscopic repair of CSD without processing scar resection avoids suturing the two edges of uterine wall together, which can reduce the difficulty of surgery. More importantly, the residual myometrium thickness can be tangibly increased. During the follow-up period, increased residual myometrium thickness was achieved in all 40 patients of Group A. However, our study’s limitation include that the number of patients is limited. Among the 121 patients, 33 patients had a CSD with residual myometrium thickness larger than 3 mm and undergone hysteroscopy treatment. While, the other 12 patients who undergone laparoscopic surgery were still not enrolled in this study, because, 8 patients disagreed to attend the research study and 4 patients did not get in touch during the follow-up. Hence, only 76 patients were enrolled in this study. The 76 operations were performed by the same surgeon, who has 20 years of clinical experience in minimally invasive gynecologic surgery, to avoid the artificial influence and experience differences. No statistical analysis of fertility outcome between the two groups summarized is another limitation. Generally, subsequent pregnancy outcome should be an assessment criterion, but less patients got pregnancy in Group B because the patients was urged to use contraception for at least 12 months after the intervention to allow the newly formed uterus scar to heal properly. Afterwards, we will provide more studies on this novel method to CSD repair, for example, the effectivity on larger scale numbers patients, the effect of subsequent fertility outcome, and so on.

## Conclusion

In summary, laparoscopic repair of CSD with retention of the integrated cesarean uterine scar may be a promising alternative to resection of CSD. This novel method can thicken residual myometrium and improve postmenstrual bleeding at short-time follow up, which may have the benefit of shorter contraception times, but more studies with long term outcome are needed.

## Data Availability

The data used and analyzed during the current study are available from the authors on reasonable request.

## Abbreviation

CSD: Cesarean scar defect
